# Profiling low-proficiency science students in the Philippines using machine learning

**DOI:** 10.1057/s41599-023-01705-y

**Published:** 2023-05-03

**Authors:** Allan B. I. Bernardo, Macario O. Cordel, Marissa Ortiz Calleja, Jude Michael M. Teves, Sashmir A. Yap, Unisse C. Chua

**Affiliations:** De La Salle University, Manila, Philippines

**Keywords:** Education, Psychology

## Abstract

Filipino students’ performance in global assessments of science literacy has always been low, and this was confirmed again in the PISA 2018, where Filipino learners’ average science literacy scores ranked second to last among 78 countries. In this study, machine learning approaches were used to analyze PISA data from the student questionnaire to test models that best identify the poorest-performing Filipino students. The goal was to explore factors that could help identify the students who are vulnerable to very low achievement in science and that could indicate possible targets for reform in science education in the Philippines. The random forest classifier model was found to be the most accurate and more precise, and Shapley Additive Explanations indicated 15 variables that were most important in identifying the low-proficiency science students. The variables related to metacognitive awareness of reading strategies, social experiences in school, aspirations and pride about achievements, and family/home factors, include parents’ characteristics and access to ICT with internet connections. The results of the factors highlight the importance of considering personal and contextual factors beyond the typical instructional and curricular factors that are the foci of science education reform in the Philippines, and some implications for programs and policies for science education reform are suggested.

## Introduction

Global concerns such as the ongoing COVID pandemic and climate change crisis underscore the importance of science and technology for providing sustainable and responsible strategies for global development. Yet in many parts of the world, students’ interest and achievement in science continue to decline (Fensham, 2008). The Philippines is one of those countries where students are observed to have low levels of science literacy for many years now (Martin et al., 2004; Talisayon et al., 2006). This pattern was confirmed when the Philippines participated for the first time in the Program for International Student Assessment (PISA) in 2018, where the results found Filipino 15-year-olds near the bottom of the ranking among 78 countries and territories (Organisation for Economic Cooperation and Development [OECD], 2019a, 2019b). Some Philippine studies have tried to understand low science achievement by looking at the curriculum (Belmi and Mangali, 2020; Cordon and Polong, 2020) and instruction (Sumardani, 2021). In this study, we used machine learning approaches to determine the most accurate predictive models that can identify the poorest-performing science students in the PISA 2018 sample. For the variables in the predictive model, we consider a range of variables in the student questionnaire of PISA that refer to the student’s home and family background, beliefs, goals, attitudes, perceptions, and school experiences. We focus on non-instructional and non-curriculum variables with the view of understanding the variables that identify the Filipino students who are most vulnerable to poor science learning.

## Filipino students’ science literacy in PISA

The Philippines participated in PISA for the first time in 2018, with students’ answering the assessments in reading, mathematics, science, and global competencies. For science literacy assessment, the PISA 2018 Framework broadly defines science literacy as “the ability to engage with science-related issues, and with the ideas of science, as a reflective citizen” (OECD, 2019a, 2019b, p. 100). According to the PISA science framework, scientific literacy relies on a combination of knowledge and competencies that are applied to different contexts. Student performance was reported using seven levels of proficiency, with Level 6 being the highest level of proficiency and Level 2 as the minimum level of proficiency. Students who achieve Level 2 proficiency are able to demonstrate the ability to use basic or everyday knowledge to explain scientific phenomena in familiar contexts and to interpret simple data sets. This level of proficiency serves as a baseline or minimum evidence for science literacy.

There were 7233 15-year-old Filipino students who participated in the PISA 2018 cycle (OECD, 2019a, 2019b), where the Philippines ranked as one of the poor-performing countries in science. The country had an average score of 357 which is significantly below the OECD average score of 489 with boys and girls performing similarly (355 and 359 average performance, respectively). Only about 22% of these students achieved Science Literacy scores at Level 2 or higher. In comparison, an average of 78% of students from OECD countries reached Level 2 or higher in the science literacy assessment. Students at Level 2 or higher can recognize the correct interpretation for familiar scientific phenomena and can use such knowledge to identify, in simple cases, whether a conclusion is valid based on the data provided. The poor performance of Filipino students is reflected in the fact that around 77% of them did not reach the minimum proficiency level. At the lowest proficiency levels (1A and 1B), students are only able to use everyday content and procedural knowledge to explain simple or familiar phenomena. Their ability to understand data and to design scientific inquiry is highly limited (OECD, 2019a, 2019b).

The pattern of Filipino students’ performance in PISA 2018 matches their achievement in another international assessment, the Trends in International Mathematics and Science Study (TIMSS). Similar to PISA, TIMSS measures students’ ability to apply their knowledge in different content areas of science. Performance was evaluated using benchmarks, each with a corresponding scale score: Low (400), Intermediate (475), High (550), and Advanced (625) (Mullis et al., 2020). Fourth-grade Filipino students who participated in the TIMSS 2019 cycle achieved an average scale score of 249, the lowest in 58 participating countries with an overall average score of around 491. Only 19% of Filipino students achieved scores in the Low benchmark or higher, which implies that the overwhelming majority of Filipino students “show limited understanding of scientific concepts and limited knowledge of foundational science facts” (Mullis et al., 2020, p. 107).

Such consistently poor achievement levels in science are very likely the results of a wide range of interacting factors. Previous research using PISA data has attempted to identify important factors that differentiate the performance of high and low high and low scorers in PISA. For example, to determine which factors contribute to the gap between high and low PISA science scores, Alivernini and Manganelli (2015) considered factors coming from country, school, and student levels. They applied a classification and regression tree analysis to the PISA 2006 data from 25 countries to identify the factors that predicted high (above Level 4) or low (below Level 2) proficiency. The strongest country-level predictor was teacher salary. At the school level, parental pressure on the school’s standards (for low teacher salaries) and school size (for high teacher salaries) predicted students’ PISA performance. At the student level, science self-efficacy and awareness of environmental issues determined whether a student would be a low or high performance in the PISA science assessment.

In this study, we employ a similar approach to studying the variables that might explain the poor performance of most Filipino students. We compare the group of poor-performing students with the group of better-performing students and consider variables related to the student’s family/home backgrounds, beliefs, goals, attitudes, perceptions, and school experiences. Instead of using statistical approaches, we use machine learning approaches to test models that best identify and distinguish the group of poor-performing students from the better-performing ones. Machine learning approaches have been proposed as complementary to statistical approaches (Lezhnina and Kismihok, 2022), particularly for purposes of handling very large numbers of variables in high-dimensional datasets (like those in the PISA) while avoiding convergence problems and for developing multidimensional complex models that may feature nonlinear relationships (Hilbert et al., 2021; Yarkoni and Westfall, 2017). Such machine learning approaches have been used to study science achievement in PISA 2015 (Chen et al., 2021), but the study focused on identifying the top performers, not the poor performers. Such approaches have been used to study the PISA 2018 data in other countries like China (Lee, 2022), Singapore (Dong and Hu, 2019), and the Philippines (Bernardo et al., 2021, 2022), but these studies focused on predicting either performance in reading, mathematics, or the average across domains, and none so far, have focused on the PISA 2018 science results. The analytic approaches are discussed in the methods section. But we first consider the range of possible predictor variables suggested by the relevant literature and that were available in the PISA student questionnaire the Filipino students answered.

## Predictors of science learning and achievement

Most studies on science education in the Philippines have focused on curriculum (Balagtas et al., 2019; Ely, 2019; Morales, 2017b), knowledge, beliefs, and practices of science teachers (Bug-os et al., 2021; Macugay and Bernardo, 2013; Orbe et al., 2018; Walag et al., 2020), and beliefs and perceptions of science learning (Alonzo and Mistades, 2021; Bernardo et al., 2008; Magalong and Prudente, 2020; Montebon, 2014); typically such studies do not empirically establish any relationship with Filipino students’ science learning or achievement. But there are some studies that do identify some predictors of Filipino students learning and achievement in chemistry, biology, physics, or some specific science lessons. And these typically fall into two types of inquiries: (a) those that investigate the learning outcomes of particular instructional strategies (Antonio and Prudente, 2021; Francisco and Prudente, 2022; Magwilang, 2016; Morales, 2016, 2017a; Orozco and Yangco, 2016), and (b) those that looked into student motivations and other non-cognitive student level variables as predictors of learning and achievement (Bernardo, 2021; Bernardo et al., 2015; Ganotice and King, 2014; King and Ganotice, 2013, 2014). In this study, we worked with variables from the student self-report questionnaire of PISA 2018, so we could not study instructional strategies (i.e., the first set of studies above), but we are able to study student-level variables similar to the latter group of studies that include motivation, self-beliefs and a host of other variables that relate to students family and home backgrounds, perceptions and attitudes related to their classroom and school experiences, and their goals and aspirations for after they finish high school. We consider what the research literature suggests about such variables below, starting with student-level variables that were included in the PISA 2018 self-report survey and that were found to be important predictors of science literacy in previous PISA research in different countries.

### Student factors

Certain student characteristics have been shown to influence their performance in science or scientific literacy. Gender appears to be associated with scientific literacy, with boys performing better than girls in the 2015 PISA cycle (OECD, 2016), but the results of numerous other studies are mixed (Cutumisu and Bulut, 2017; Lam and Lau, 2014; Sun et al., 2012). Affective and motivational factors seem to be important correlates of science achievement in PISA; these factors include students’ enjoyment of science and perceived value of science (Ozel et al., 2013), positive motivations, interest, more sophisticated epistemic beliefs (Hofverberg et al., 2022; She et al. 2019), self-efficacy, intrinsic and instrumental motivations for learning science (Kartal and Kutlu, 2017; Mercan, 2020), having a growth mindset (Bernardo, 2021, 2022; Bernardo et al., 2021), among others. Other motivation-related processes are also associated with science literacy achievement. These include students’ projective self-assessments of their own abilities and their future aspirations (Lee and Stankov, 2018), perseverance and willingness to solve problems (Cutumisu and Bulut, 2017), and use of metacognitive strategies (Akyol et al., 2010; Callan et al., 2016). Interestingly, students’ reading skills and reading strategies have also been associated with science achievement (Barnard-Brak et al., 2017; Caponera et al., 2016). The role of reading strategies is proposed to be important as science learning depends to an extent on students’ comprehension of scientific text (Cano et al., 2014; Kolić-Vrhovec et al., 2011) and this association seems particularly important when the students are learning science in a second language instead of their home language (Van Laere et al., 2014), which is the case with the Filipino students who participated in PISA 2018.

### Family and home factors

The socioeconomic status (SES) of students’ families has been a consistent predictor of scores in PISA (Lam and Zhou, 2021; Lee and Stankov, 2018), and this is true in the domain of science (Sun et al., 2012). This variable has been unpacked and many other factors associated with SES have been identified as predictors of achievement in PISA. These SES-related factors include the educational attainment and occupation of their parents (Chen et al., 2021; Schulze and Lemmer, 2017). In one such study, researchers found that parents’ education had the largest indirect effect on children’s PISA test scores (Burhan et al., 2017). The influence of each parent’s education, however, appears to differ. A study that analyzed the PISA 2000 performance of 30 countries found that the mother’s educational attainment had a greater impact on students’ scores than the father’s educational attainment (Marks, 2008). Similar to education, parents’ occupations also predicted students’ learning outcomes. Students whose parents had a higher level of occupation were found to have higher scientific competencies than students whose parents were low-skilled workers (Chi et al., 2017). Another variable related to SES is the students’ access to information and communication technologies (ICT) at home, particularly ICT with access to the Internet. ICT availability and use positively predicted performance in various PISA assessments (Hu et al., 2018; Petko et al., 2017; Yoon and Yun, 2023). We also note that studies indicate SES seems to be associated with some student-level factors. For example, SES is strongly associated with feelings of school belonging (King et al., 2022).

Other than SES-related factors at home, parental involvement and family investment in children’s education also appear to influence students’ academic performance (Ho and Willms, 1996). Using data from a national survey of Chinese students’ science literacy, Wang et al. (2012) found that students’ high scores were associated with parents’ investment in their children’s education through the purchase of educational materials and other resources at home. A study of ninth-grade students in South Africa found that family experiences, such as the learning environment at home, were related to the student’s motivation to learn science (Schulze and Lemmer, 2017).

### School factors

The school characteristics that have been shown to influence students’ scientific literacy performance include SES (or SES composition), school enrollment size, and location. Wang et al. (2012) found that certain school characteristics, namely school standing, having libraries and computer laboratories, good relationships between teachers and students, and funding for teacher training were associated with higher science literacy scores. School SES composition was found to be strongly associated with high scientific literacy scores of Australian students (McConney and Perry, 2010). Analysis of Hong Kong students’ PISA scores revealed that school SES composition partly explained differences in science achievement (Sun et al., 2012). Class size (Bellibaş, 2016; although see Lam and Lau, 2014) and school location (Topçu et al., 2014) are also predictors of science achievement.

Other than these school characteristics, students’ experiences and perceptions of their classroom and school environments also predict their achievement in PISA. In a study of the performance of Chinese students in the 2015 PISA, Huang (2020) found that reported experience of bullying in school was associated with achievement scores, and this relationship was medicated by the student’s sense of belonging in school. School disciplinary climate and quality of student-teacher relationship were significant predictors in particular countries (Shin et al., 2009); with the effect of disciplinary climate possibly having a more positive impact on students from low SES groups but the evidence across countries is mixed (Chi et al., 2018; Scherer, 2020).

## The current study

The extant literature suggests that a wide range of factors at the student, family/home, and school level are likely predictors of science literacy, although some of these factors were shown to be important predictors in some countries but not all. In this study, we explore a range of such factors to inquire which best identifies the poor-performing Filipino students in contrast to the better-performing ones. The factors explored in the study are among those in the PISA 2018 student self-report survey that Filipino students answered.

Most education research that examines relationships among such variables applies statistical approaches. In such studies, correlations can show the linear relationship between each variable of poor and better-performing groups. In the current Philippines PISA 2018 dataset where we examine 85 variables as predictors, the possible correlations are over 7000 in number. For a more complex, nonlinear system with hundreds of variables that are not independent, we believe that it is best to use machine learning models. In contrast to the standard statistical approach, machine learning models capture the high-dimensional, possibly nonlinear, interrelations among a very large number of predictors (Hilbert et al., 2021; Yarkoni and Westfall, 2017), while identifying those most relevant to prediction. And, in order for the analysis to be more valid, we argue that the model should be optimal, in this case, the model with the best accuracy. For this study, we try out different machine-learning approaches to determine the best model to uncover the relationships between these variables.

The specific objective is to use machine learning approaches to determine the most accurate model that best identifies the Filipino students who performed at the lowest levels in the science domain of PISA 2018. We sought the best model that will indicate the factors that identify the students who are vulnerable to poor learning in science in the hope that the model will call the attention of Filipino educators to the non-instructional and non-curricular factors that contribute to poor learning in science among Filipino learners. The variables that were considered included *student factors* (e.g., motivations, self-beliefs, goals, aspirations), *family/home factors* (e.g., family SES, parents’ education and occupations, learning resources at home), and *classroom/school factors* (e.g., instruction time for science, teacher behaviors, perceived school environment, self-reported social experiences in school).

## Method

**Fig. 1:**
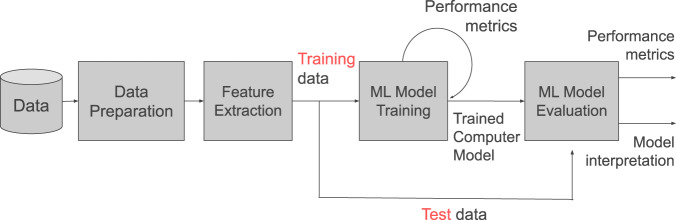
The data processing methodology is generally composed of data preparation and machine learning (ML) modeling. To find the optimal computational model, the whole data processing pipeline is performed for different sets of hyperparameters, for different machine learning approaches.

Our methodology for determining the best model that features the most important variables that identify the low-performing Filipino student in science is summarized in Fig. 1, which shows the different phases of our data analysis. The first step is data preparation which entails data cleaning, that is, removal of variables with 100% missing data, identification, and imputation of entries with missing values, and variable scaling. Next is feature selection which involves the careful refinement of the list of variables that may contain negative suppressors. Then, machine learning model training follows to search for the best nonlinear prediction model. Finally, the machine learning model evaluation describes quantitatively the model performance and reports variable importance.

### The dataset

The data we used in the analysis were from the Philippine sample in the PISA 2018 data (publicly accessible at https://www.oecd.org/pisa/data/2018database/). PISA 2018 test items for the science subject measure the ability to engage with science-related issues as a thoughtful citizen (OECD, 2019a, 2019b). To assess this, the questions given are related to *contexts*, e.g. personal, local and global issues, both current and historical that require understanding in science and technology; to *knowledge*, e.g. content, procedural, and epistemic; and to *competencies* that exhibit the ability to explain phenomena scientifically, evaluate and design scientific inquiry, and interpret data and evidence scientifically. In addition to these, students answered background questionnaires about themselves, their homes, and the school and learning experiences. As discussed, these variables were considered in this study. The performance of students is estimated and reported as 10 plausible values with 0.88 reliability for the Philippine science data.

A two-stage stratified sampling design was followed to obtain the nationally representative sample: (a) 187 schools were randomly selected from the country’s 17 regions, with the number of schools selected proportional to the regional distribution of schools, (b) students were then randomly selected from each school. As mentioned in the introduction, the final sample was 7233 15-year-old students. From the database, we identified 85 variables that referred to student, home/family, classroom/school factors suggested by the extant literature as possible predictors of science literacy, which we measured using the first plausible value of science literacy (PV1SCIE).

### Data preparation

**Fig. 2:**
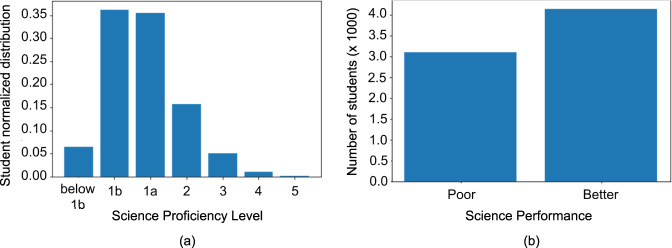
Summary of distribution of students in different science proficiency categories. Normalized Science proficiency level distribution of students (**a**) and distribution of students with *poor* and *better* performance in science (**b**). *Poor* performance category is for those students who belong to Level 1b and below proficiency levels, otherwise, the students are assigned to the *better* performance category.

As reported earlier, over 80% of the Filipino students who participated in PISA 2018 were found to have less than Level 2 proficiency in science. The detailed distribution of participants across the different proficiency levels is shown in Fig. 2a. Because our goal is to identify the variables that are potentially influential in identifying the extreme poor performers in science, we decided to train a binary classification model that identifies these students and to study the variables that are important in this model prediction. For the binary classification, the sample data was divided into two categories; the (1) poor-performing students, who have proficiency at Level 1b and below, and (2) better-performing students, who have proficiency at Levels 1a, 2, and above. The data distribution for the two groups is shown in Fig. 2b.

The samples were further trimmed down as part of the data preparation. Students with more than 50% of the total variables missing were dropped from the dataset, obtaining the final data distribution in Table 1. Sampled randomly, around 80% of the data were used as the training samples for the model training while the unseen or remaining data were used as the test data.

**Table 1:** Data distribution of train and test sets with 80–20 split.

Data split	Poor performance (Level ≤ 1b)	Better performance (Level ≥ 1a)	Total
Training data	2419	3297	5716
Test data	628	801	1429
Total	3047	4098	7145

Note the imbalance in the number of training samples for the good and poor-performing students.

To avoid bias in training the model, data balancing was conducted by *oversampling* the poor-performance samples and *undersampling* the better-performance samples. For the poor-performing category with 2419 samples, the Synthetic Minority Oversampling Technique or SMOTE algorithm (Chawla et al., 2002) was applied to increase the samples. The SMOTE first chooses a random sample from the minority class, for example, sample A. Then, it looks for its nearest neighbor of the same class, for example, sample B. The algorithm performs a convex combination of the two samples to produce the synthetic sample. For the better-performing category with 3297 samples, the Tomek Links (Tomek, 1976) algorithm is used to undersample the majority class. The algorithm removes the ambiguous samples from the majority class, which is the data from the majority class that is closest to a minority class data. The final number of training samples for each of the poor and better performance categories is 3214.

The list of variables was further refined by removing variables with 100% missing values (i.e., the questions were not included in the set of questions asked for Filipino students). Those remaining variables with missing values were imputed using the *k*-nearest neighbor algorithm, where *k* = 7. Also, initial experiments showed the occurrences of negative suppressors. To minimize the number of suppressors, we removed variables with high correlation with other factors, i.e. |*⍴*| > 0.75. Finally, normalization by scaling was performed per variable. In summary, 13 variables were removed because these variables have missing values only and 20 variables were removed because they have high correlation with other variables. The final number of variables is 72 plus the scientific literacy score of PV1SCIE.

### Machine learning modeling

Our approach to determining the key variables that identify Filipino students with poor performance in science used machine learning, aiming to come up with a computational model that relates the input variables to the target variables. The design for the computational model is evaluated in terms of training and test accuracy to measure the model performance in both seen and unseen data and the Area under the Region-of-Convergence curve (AUC) to determine how well the separation of data is in the model space.

**Fig. 3:**
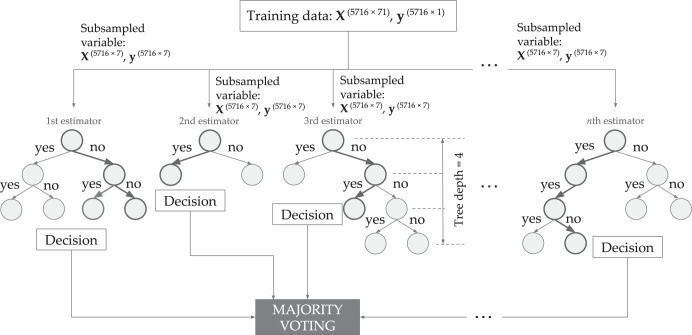
The designed Random Forest model for classifying poor and better-performing students given a set of 71 variables. It is composed of *n* = 500 decision trees called estimators with a maximum tree depth of 20. Each input to the estimator uses only a subset of variables equal to ceil(log_2_71) or 7 variables. This minimizes the model overfitting due to the original large number of variables.

Exhaustive hyperparameter search on the following computational models: support vector machines (SVM), logistic regression, multilayer perceptron (MLP), decision tree, and random forest (RF). Performance in terms of accuracy revealed that the best model is the RF classifier, having 500 estimators, maximum decision tree depth equal to 20, and maximum features equal to ceiling(log_2_71) = 7 variables per individual tree, which is the best classifier. (Please refer to Supplementary File for the performance summary of the machine learning models considered.) To illustrate the RF model, please refer to Fig. 3. The RF model is composed of several independent decision trees that are trained independently on a random subset of data. To measure the quality of a split, *entropy* is used to measure the information gain.

## Results

### Model performance

**Fig. 4:**
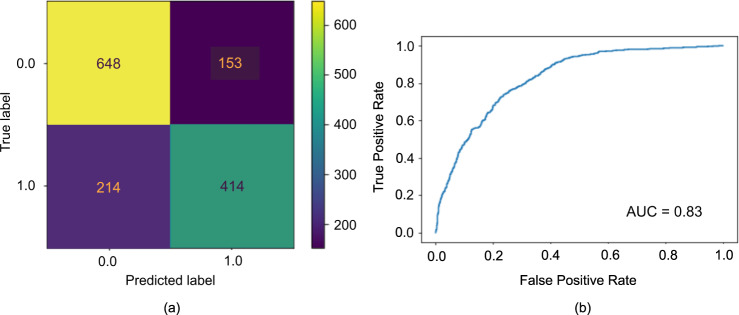
Summary of accuracy and precision performance results for Random Forest model. The confusion matrix (**a**) and the ROC (**b**) summarizing the performance of the RF model in classifying the PISA 2018 Science Proficiency of Filipino students. The average accuracy is 0.74 and the area under the ROC being equal to 0.83.

The summary of the model performance is shown in Fig. 4. The positive class for this study refers to the poor-performing class while the negative class refers to the better-performing class. Since the test dataset is not balanced, three performance metrics were observed: classification accuracy, precision, and recall. Accuracy is the ratio of correctly classified students, whether poor-performing or better-performing students, over the total number of students. Precision is defined as the ratio of the number of correctly predicted poor-performing students and the total number of predicted poor-performing students. Recall is the ratio of correctly predicted poor-performing students and the total number of poor performing. High precision and recall show that the model is returning accurate results (high precision), and returning a majority of all positive results (high recall).

**Fig. 5:**
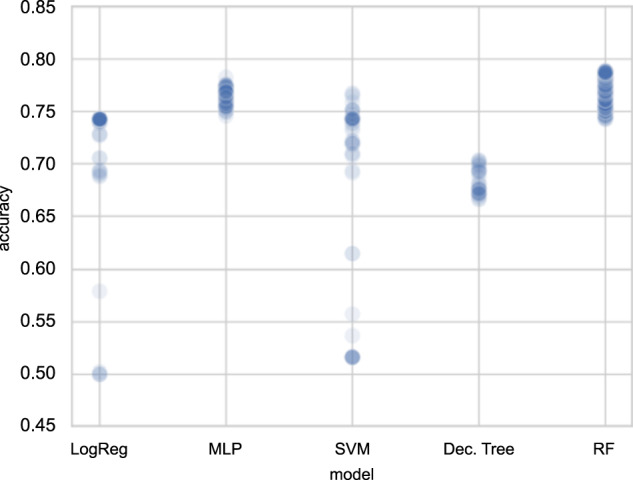
Summary of grid search accuracies of the different machine learning models. The scatterplot illustrating the range of test accuracies during the cross-validation on best machine learning models shows that the RF returned the best accuracies.

The RF Classifier returned a good balance of precision and recall on the training data with values equal to 0.74, and 0.79, respectively. In addition to this balance, among the different classifiers considered, the grid-search accuracy (see Fig. 5), shows that the RF classifier returned the best performance with final accuracy equal to 0.74, considering the precision and recall balance. The final precision, recall and accuracy using the test data are 0.73, 0.66, and 0.74, respectively. The area under the receiver operating curve (AUC) is 0.83 which implies a fairly good-fit model. A perfect classifier has AUC = 1.0 which implies that the model was able to separate the two classes, i.e. positive and negative, of data. The worst classifier, i.e. chance level accuracy, has AUC = 0.5.

### Model Interpretation

**Fig. 6:**
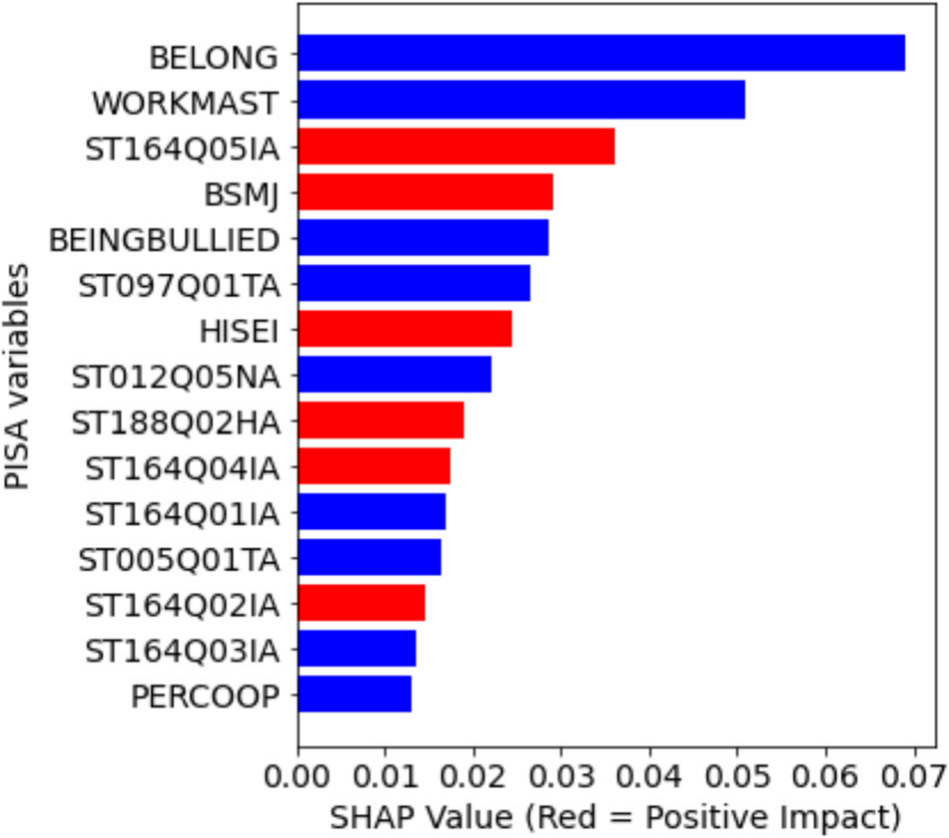
The top 15 variables that are important in predicting the Science proficiency (poor or better) of Filipino students in PISA 2018, listed in decreasing importance (SHAP value) from top to bottom. Blue bars represent variables that negatively affect the prediction of poor-performing students while red bars indicate that a variable positively affects the prediction of poor-performing students.

We investigated the feature importance learned during training by the RF classifier. We used Shapley additive explanations (SHAP) which is a scheme based on cooperative game theory to interpret the contributions of features in the prediction. For the RF classifier in this study, these top 15 key features or variables are shown in Fig. 6.^1^ The important variables can positively affect or negatively affect the prediction of poor performance class (*y* = 1). Particularly, one student with higher values for the variables BELONG, WORKMAST and BEINGBULLIED will negatively affect the prediction of identifying the poor performers in science. Similarly, for students with high ST164Q05IA, BSMJ, and HISEI values, the prediction of identifying poor performers in science is higher since these values positively impact this prediction. We describe the 15 variables in meaningful groupings below.

The largest cluster of variables relates to students’ metacognitive awareness in reading or their perceptions about the usefulness of particular metacognitive strategies when reading texts in their classes. These variables are related items, where students were asked to indicate whether the indicated strategy is useful for understanding and memorizing the texts they read. Three of the variables positively identified the poor-performing students: (ST164Q05IA) “I summarize the text in my own words,” (ST164Q04IA) “I underline important parts of the text,” and (ST164Q02IA) “I quickly read through the text twice.” These three reading strategies involve relatively low metacognitive skills and are often ineffective, and poor-performing Filipino science students tend to see them as useful. On the other hand, two of the variables negatively identified poor-performing students: (ST164Q01IA) “I concentrate on the parts of the text that are easy to understand,” and (ST164Q03IA) “After reading the text, I discuss its content with other people.” The poor-performing Filipino science students tend to perceive these strategies as not useful.

The next largest cluster of variables relates to the student’s classroom and school experiences. Sense of belonging (BELONG) and perceived cooperation among students (PERCOOP) both negatively identify poor-performing students; that is, students who perform poorly in science report a low sense of belonging and perceive less cooperation among students. These two variables suggest negative social relations experienced by poor performers in science. Fortunately, self-report of experiencing bullying (BEINGBULLIED) was also negatively identified as the poor performers in science, so they tended to report less experiences of bullying in school. The last variable related to classroom experiences was how often “Students don’t listen to what the teacher says” (ST097Q01TA), which negatively identified the poor performers in science. The poor-performing science students were less likely to say that students often do not listen to the teacher. We should clarify that the item refers to teachers who use English in their classes, which refers to teachers in several subjects including science, mathematics, and English.

Three variables relate to the students’ affective or motivational experiences. The student’s motivation to master assigned learning tasks (WORKMAST) negatively identify poor-performing students, which means they tend to have low work mastery motivation. On the other hand, the student’s expected occupational status (BSMJ) and feeling proud about the things they accomplished (ST188Q02HA) both positively identified the poor-performing students. So the students who scored very low scores in science also tended to report higher job aspirations and being proud of their accomplishments compared to others. It seems that the student’s low achievement in science is unrelated to their future occupational plans and their present sense of accomplishment.

Finally, the remaining variables relate to the student’s family and home learning resources. Having smartphones with internet access at home (ST012Q05NA) negatively identified the poor-performing students, which means they were less likely to have this learning resource. But interestingly, the mother’s education (ST005Q01TA) negatively identified the poor-performing students, but the parents’ occupational status (HISEI) positively identified the students. This means that having mothers with lower educational attainment but having parents with high-status occupations also identified the students who were performing poorly in science. We could be seeing a pattern where low achievement in science is probably not viewed or experienced as a hindrance to higher-status professions. We explore this point and other results in the discussion section.

## Discussion

We used machine learning approaches to explore the best model for identifying the poorest-performing Filipino students in science using the PISA 2018 data. The Random Forest model was found to have the highest accuracy performance and the SHAP analysis indicated 15 variables that identified the poorest-performing science students.

### Caveats and limitations

Before we discuss the meaning and implications of the details of the results, we need to underscore some important limitations in our study. First, our study cannot speak to the instructional and curricular factors that are typically the subject of discussions on improving science education in the Philippines. Second, the predictors in the model were limited to the variables in the PISA student self-report questionnaire. While there was a wide range of variables in the student questionnaire, many of the questions referred to reading (because the 2018 cycle of PISA was focused on reading), and thus, could not be included in our study. We also did not include variables from the school-head questionnaire about school characteristics, resources, and practices; nor could we include other potentially important predictors of science achievement that were not included in the PISA. Thus, there are possibly other variables that identify poor-performing students that are beyond the scope of this inquiry.

One important caveat relates to the predictive nature of the machine learning approaches, which treat variables equally without any theoretical presuppositions. Machine learning approaches focus on prediction accuracy and is not used to test explanatory models that specify theoretical relationships among variables (Shmueli, 2010; Yarkoni and Westfall, 2017). As such, the variables identified in the most accurate model may not have any obvious theoretical connection. These caveats notwithstanding, there are useful insights revealed by the analysis, which we discuss below.

### Reading strategies for learning science

Metacognitive awareness regarding five different strategies identified the poor performers in science. It may seem surprising that reading strategies play an important role in identifying poor science performers, but the results make sense if one considers that much of science learning might be based on reading science textbooks (instead of doing laboratory experiments or field projects). Research with Italian students, for example, showed a difference in science achievement between good and bad readers, regardless of whether the science items involved low or high reading demand (Caponera et al., 2016). There were similar associations between reading comprehension and science achievement in a study of Spanish (Cano et al., 2014) and Filipino students (Imam et al., 2014). We note that our results do not actually involve reading comprehension, but metacognitive awareness of reading strategies, similar to a study of Croatian students that established a relationship between students’ reading strategies and comprehension of scientific texts (Kolić-Vrhovec et al., 2011). It is plausible that poor achievers in science are those that might be adopting the wrong reading strategies in reading their science textbooks.

### Families’ and students’ resources and aspirations

High social, cultural, and economic resources in the students’ families (Lam and Zhou, 2021; Sun et al., 2012) and higher professional aspirations (Lee and Stankov, 2018) are typically associated with better achievement. But in our results, the poor-performing students were identified by higher job aspirations and stronger pride about one’s achievements. It is as if low achievement in science was not a consideration when students think about their future occupations nor when they assess their self-worth and pride. If we consider that the lower educational attainment of the mothers and higher occupational status of parents also predicted the poor-performing, it may be that students view their poor achievement in science as not relevant to their future occupational prospects, as their parents enjoy good occupations, even if their mothers are not highly educated. This interpretation asserts that science achievement might not be valued in pragmatic terms by the students based on what they see in their elders, which might also explain the role of low work mastery in school in identifying poor-performing students. Indeed, it is possible that many high-status occupations in the Philippines do not require knowledge of science, and as such, persevering and doing well in science might not be an important motivation among the students. This interpretation will need to be verified in future studies.

### Negative social experiences

It was interesting to note that experience of bullying was a negative factor in the model, so it was not the case that experience of bullying was positively linked to poorer science achievement, as was found in Chinese students (Huang, 2020). However, two factors that indicate relational issues in school are identified with the poor performers: reporting a low sense of belonging and low cooperation among students in school. These factors suggest that a lack of connectedness and a collective spirit might be associated with poor science performance. Trinidad (2020) found that school-level and student-level measures of school climate were predictors of Filipino students’ mathematics achievement; such social factors might also have similar roles in Filipino learners’ poor science achievement.

### Access to ICT for learning

One factor that may be increasingly important in identifying poor science achievers is access to ICT devices with internet access. Studies on Filipino students; PISA achievement in reading (Bernardo et al., 2021) and mathematics (Bernardo et al., 2022) also found the same factor as a predictor of achievement, consistent with much of the research in other countries (Hu et al., 2018; Petko et al., 2017; Yoon and Yun, 2023; but see Bulut and Cutumisu, 2018). Presumably, access to the internet outside the school environment has become an important resource for learning science; perhaps not just for accessing relevant scientific knowledge available online but also as a means of communicating with classmates for information sharing, collaboration in learning activities, and supporting each other’s motivations and engagement in science learning. Filipino students without such access are disadvantaged in the domain of science.

### Practical implications: Focusing on the lowest achievers

The current study provides some analysis that could inform reform efforts in the domain of science learning, particularly as it concerns the lowest-achieving Filipino students in science. The results and discussion focus on factors that seem to characterize these lowest-achieving science students, and as such provide entry points to identifying these students and designing interventions for this particular group of students. Our approach focuses on the sizable proportion (over 35%) of Filipino students who have been assessed as demonstrating extremely low competencies (levels 1b and below) in science. The Philippine educational system does not lack programs for the more gifted students in science such as special science schools (Faustino and Hiwatig, 2012), competitions, scholarships, and other forms of support for students pursuing advanced studies and careers in science (De La Cruz, 2022). But there is not much that is documented about what is being done for the students like the 35% who are demonstrating extremely low levels of scientific literacy, even if they have progressed to the high school levels of the country’s formal education system. The first important implication of our findings is that these students need to be identified and understood before their science learnings can be addressed.

We should clarify that the characterization of poor-performing Filipino students in science should not be interpreted as the opposite characterization of better- or high-performing students. It is likely that there are qualitative differences between the experiences of poor and better science learners that are not captured by simply assuming a linear relationship between the factors that predict science learning. Indeed, if our machine learning approach was applied to identify the high-achieving students (i.e., Levels 4–6), it is likely that a different set of variables will be in the best machine learning model (and that can be explored in a different study). But by implication, the characterization of the poor-performing students in the results does not point to simple instructional or curricular interventions, and we do think there are some important policy implications that can be considered by stakeholders who are concerned with improving science education achievement among Filipino learners.

#### Instructional programs for poor achievers

Science educators have long noted that there are profound diversities in students of different ability levels, that simply assuming that one form of good teaching fits all types of learners is no longer tenable (Ault, 2010; Lynch, 2001; Yang et al., 2019). In this regard, the science education reform community of stakeholders should consider moving away from a one-size-teaching-fits-all approach that tends to be designed for students in the middle range of abilities using whole class instruction, and instead, move towards approaches that consider diverse adaptive learning approaches (Yang et al., 2019) and differentiated instruction (Pablico et al., 2017) that might be more responsive to (or at least that might not simply ignore) the needs of the low achievers.

#### Ensuring reading skills

There is a lot of evidence that good reading strategies and reading comprehension are strongly associated with science achievement (Cano et al., 2014; Caponera et al., 2016; Kolić-Vrhovec et al., 2011), but Filipino learners on average have extremely poor reading skills in English (Bernardo et al., 2021), which is the medium-of-instruction in science. Presumably, there are science learning activities that are more experiential and discovery-oriented and less dependent on students’ reading textbooks; but a previous study of students’ perception of science classes revealed a trend of decreasing science inquiry activities accompanied by an increase in self-learning, presumably involving reading textbooks and learning modules from Grade 5 to 10 (Bernardo et al., 2008). If Filipino science learners will be expected to do much of their learning through textbooks and learning modules in English, there should be strong efforts to strengthen the reading strategies and skills of Filipino learners.

#### Science in future professions and Philippine society

We interpreted part of the results as being associated with the view that science learning and achievement are irrelevant to higher future occupational aspirations. While these interpretations are speculative, there is probably a strong basis for the view that one does not need science to attain respectable occupations in the Philippines. There are many models of successful Filipino professionals and individuals who do not seem overtly display knowledge and use of science. In this regard, efforts to improve the science achievement of Filipino students might need to reckon with the perceived irrelevance of science in Philippine society. Scholars have problematized the lack of a science culture in the Philippines (Pertierra, 2004), perhaps vividly displayed in the recent COVID-19 pandemic, when there was widespread uncritical sharing of misinformation on vaccines, false cures, and other scientific matters through social media and social networks (Amit et al., 2022) and when scientific advice on pertinent issues was diluted and filtered before decisions were made by national leaders (Vallejo and Ong, 2020). Beyond schools, there should be efforts to change public perceptions of the importance of science in Filipinos’ social mobility and Filipino society’s development.

#### Improving school climate

The poor-performing students in science were identified by reports of a low sense of belonging in school and low perceived cooperation among students. These social experiences may be associated with lower achievement as they indicate a lack of meaningful sense of connectedness with students and teachers in the school, which is associated with lower engagement in the science classes, even if the social experiences are not specifically confined or referring to the science classes. The factors that contribute to these negative social experiences might vary across schools and communities and should be understood in proper contexts. Once the nature and causes of these social experiences are better understood, appropriate contextualized interventions can be developed.

#### Access to ICT devices and connectivity

Previous studies have documented how ICT availability and use positively predicted student achievement (Hu et al., 2018; Petko et al., 2017; Yoon and Yun, 2023), and similar results were also found in Filipino students’ achievement in reading (Bernardo et al., 2021), mathematics (Bernardo et al., 2022), and now in science. Together with improving access to the internet, there should be an effort to train teachers and students how to more effectively use the internet to deepen their learning of science concepts and processes, and in ways that adapt to students’ diverse abilities, interests, motivations, and circumstances (Yang et al., 2019).

## Conclusions

Based on the assumption that science-for-all requires all Filipino citizens to acquire the scientific literacy required to effectively engage with and contribute to Philippine society in the 21st Century, we focused on the Filipino students with the lowest levels of science achievement in PISA 2018. We used machine learning to explore the variables that best identify the poor-performing Filipino students, as these variables could be used to better track and understand their learning needs. Our study points to a cluster of variables related to the student’s reading strategies, occupational aspirations, social experiences in school, and access to ITC and the internet. The variables depart from the typical focus of reform efforts on teachers’ competencies, curriculum, and instruction. But if we truly want to improve Filipino students’ science literacy, we need to understand the experiences of students who are failing to do so, as these point to problems that need to be addressed in their learning experiences in Philippine schools.

## Supplementary information

Supplementary File

## Data Availability

The data analyzed in this study are available on the PISA 2018 Database page on the website of the Organisation for Economic Co-operation and Development at https://www.oecd.org/pisa/data/2018database/, accessed on 17 Feb 2020.

## Footnotes

1 For completeness, we also conducted a SHAP analysis for the best algorithm for each of the other machine learning approaches. A comparative summary of the top 15 variables that feature in the prediction models is shown in Supplementary File.
