# Supplementary material for: Profiling low-proficiency science students in the Philippines using machine learning
Source: Humanit Soc Sci Commun. 2023 May 3;10(1):192. doi: 10.1057/s41599-023-01705-y (PMC10154750; doi:10.1057/s41599-023-01705-y)
Supplement: Supplementary file 1 — Supplementary File [file 41599_2023_1705_MOESM1_ESM.pdf]

Supplementary File

Performance summary of the different Machine Learning models considered in the study. For this study, the positive and negative cases refer to the poor-performing students and better-performing students, respectively. The *precision* metric is computed as the ratio of the number of correctly predicted positive, *i.e.* True Positive (TP) samples and the total number of predicted positive samples *i.e.* TP and False Positives (FP). *Recall* is computed as the ratio of TP and the total number of positive samples (P). *Accuracy* is the ratio of the correctly predicted samples divided by the total number of samples. Results show that RF performed better in terms of precision and accuracy. Text in bold means the best performance for a specific metric.

	**Precision**	**Recall**	**Accuracy**
Logistic Regression	0.69	**0.79**	0.75
Multilayer Perceptron	0.65	0.78	0.73
SVM	0.65	0.62	0.69
Decision Tree	0.63	0.64	0.69
Random Forest	**0.74**	0.70	**0.76**

Summary table of top 15 variables that have impact on each machine learning model

Random Forest	Logistic Regression	MultiLayer Perceptron	Support Vector Machines	Decision Tree
BELONG	**ST164Q051A**	ST004D01T	ST004D01T	**BELONG**
WORKMAST	**BEINGBULLIED**	**ST005Q01TA**	**ST164Q051A**	**ST164Q051A**
ST164Q051A	ST004D01T	**BEINGBULLIED**	**WORKMAST**	SCHLTYPE
BSMJ	**ST005Q01TA**	**HISEI**	SCHLTYPE	**ST164Q021A**
BEINGBULLIED	**HISEI**	**BELONG**	ST011Q02TA	**BSMJ**
ST097Q01TA	ST164Q061A	**WORKMAST**	**ST005Q01TA**	**BEINGBULLIED**
HISEI	SCHLTYPE	**ST164Q051A**	**BELONG**	RESILIENCE
ST012Q05NA	**WORKMAST**	SCHLTYPE	HISCED	**WORKMAST**
ST188Q02HA	**BSMJ**	ST011Q02TA	ST097Q03TA	ST097Q101TA
ST164Q041A	ATTLNACT	**BSMJ**	**BEINGBULLIED**	GFOFAIL
ST164Q011A	**ST012Q05NA**	**ST012Q05NA**	ST154Q061A	SMINS
ST005Q01TA	ST011Q02TA	**ST188Q02HA**	**ST012Q05NA**	ATTLNACT
ST164Q021A	**ST188Q02HA**	ST164Q061A	ST164Q061A	ST011Q11TA
ST164Q031A	ST011Q02TA	ST188Q03HA	**ST164Q031A**	ST164Q061A
PERCOOP	HISCED	ST011Q05TA	ST188Q01HA	ESCS

Note: Red font indicates positive impact on the prediction model, blue font indicates negative impact. Variables in bold font in the last four columns indicate common variable in the Random Forest model.

The Shapley additive explanations analysis (SHAP) was used to help interpret the contributions of variables in the best performing prediction model, the Random Forest model. The logic of using the machine learning approach in the study required that the best fitting model is the only model that is used and interpreted. However, we provide results of SHAP analysis for the best model and the other four machine learning approaches. As expected, there is some overlap in the important variables (see items in bold) but there were many dissimilarities in the important variables across the models. We provide these results merely for illustration; the interpretation of the less well-performing models or of the similarities and differences across the model is not within the aims and scope of the current study.
